# Dysregulated phosphorylation of Rab GTPases by LRRK2 induces neurodegeneration

**DOI:** 10.1186/s13024-018-0240-1

**Published:** 2018-02-13

**Authors:** Ga Ram Jeong, Eun-Hae Jang, Jae Ryul Bae, Soyoung Jun, Ho Chul Kang, Chi-Hu Park, Joo-Ho Shin, Yukio Yamamoto, Keiko Tanaka-Yamamoto, Valina L. Dawson, Ted M. Dawson, Eun-Mi Hur, Byoung Dae Lee

**Affiliations:** 1 Department of Neuroscience, Graduate School, Kyung Hee University, Seoul, South Korea; 2 Center for Neuroscience, Brain Science Institute, Korea Institute of Science and Technology (KIST), 5 Hwarang-ro 14-gil, Seongbuk-gu, Seoul, 02792 South Korea; 3 Convergence Research Center for Diagnosis, Treatment and Care System of Dementia, KIST, Seoul, South Korea; 4 Division of Bio-Medical Science &Technology, KIST School, Korea University of Science and Technology, Seoul, South Korea; 5 Department of Physiology, Ajou University School of Medicine, Suwon, South Korea; 6 HuGex Co. Ltd., Incheon, South Korea; 7 Division of Pharmacology, Department of Molecular Cell Biology, Samsung Biomedical Research Institute, Single Cell Network Research Center, SungKyunKwan University School of Medicine, Suwon, South Korea; 8 Center for Functional Connectomics, KIST, Seoul, South Korea; 9 Neurodegeneration and Stem Cell Program, Institute for Cell Engineering and Department of Neurology, Johns Hopkins University School of Medicine, Baltimore, USA; 10 Department of Physiology, Johns Hopkins University School of Medicine, Baltimore, USA; 11 Solomon H Snyder Department of Neuroscience, Johns Hopkins University School of Medicine, Baltimore, USA; 12 Department of Pharmacology & Molecular Sciences, Johns Hopkins University School of Medicine, Baltimore, USA; 13 Department of Physiology, School of Medicine, Kyung Hee University, 26 Kyungheedae-ro, Dongdaemun-gu, Seoul, 02447 South Korea

**Keywords:** LRRK2, Rab GTPases, Phosphorylation, Neurodegeneration, Parkinson’s disease

## Abstract

**Background:**

Mutations in *leucine-rich repeat kinase 2* (*LRRK2*) are the most common cause of familial and sporadic Parkinson’s disease (PD). Elevated kinase activity is associated with LRRK2 toxicity, but the substrates that mediate neurodegeneration remain poorly defined. Given the increasing evidence suggesting a role of LRRK2 in membrane and vesicle trafficking, here we systemically screened Rab GTPases, core regulators of vesicular dynamics, as potential substrates of LRRK2 and investigated the functional consequence of such phosphorylation in cells and in vivo.

**Methods:**

In vitro LRRK2 kinase assay with forty-five purified human Rab GTPases was performed to identify Rab family proteins as substrates of LRRK2. We identified the phosphorylation site by tandem mass-spectrometry and confirmed it by assessing phosphorylation in the in vitro LRRK2 kinase assay and in cells. Effects of Rab phosphorylation on neurodegeneration were examined in primary cultures and in vivo by intracranial injection of adeno-associated viral vectors (AAV) expressing wild-type or phosphomutants of Rab35.

**Results:**

Our screening revealed that LRRK2 phosphorylated several Rab GTPases at a conserved threonine residue in the switch II region, and by using the kinase-inactive LRRK2-D1994A and the pathogenic LRRK2-G2019S along with Rab proteins in which the LRRK2 site was mutated, we verified that a subset of Rab proteins, including Rab35, were authentic substrates of LRRK2 both in vitro and in cells. We also showed that phosphorylation of Rab regulated GDP/GTP-binding property in cells. Moreover, in primary cortical neurons, mutation of the LRRK2 site in several Rabs caused neurotoxicity, which was most severely induced by phosphomutants of Rab35. Furthermore, intracranial injection of the AAV-Rab35 -T72A or AAV-Rab35-T72D into the substantia nigra substantially induced degeneration of dopaminergic neurons in vivo.

**Conclusions:**

Here we show that a subset of Rab GTPases are authentic substrates of LRRK2 both in vitro and in cells. We also provide evidence that dysregulation of Rab phosphorylation in the LRRK2 site induces neurotoxicity in primary neurons and degeneration of dopaminergic neurons in vivo*.* Our study suggests that Rab GTPases might mediate LRRK2 toxicity in the progression of PD.

**Electronic supplementary material:**

The online version of this article (10.1186/s13024-018-0240-1) contains supplementary material, which is available to authorized users.

## Background

Parkinson’s disease (PD) is the second most prevalent neurodegenerative disease, affecting about 1-3% of the elderly population [1]. Mutations in *Leucine-rich repeat kinase 2* (*LRRK2*) comprise the leading cause of familial PD, and genome-wide association studies have also identified *LRRK2* as a risk locus for sporadic PD [2, 3]. LRRK2 is a large multi-domain protein that encompasses a kinase domain, a GTPase domain composed of a Ras of complex (ROC) and a C-terminus of ROC region, and several protein-protein interaction domains. Multiple lines of evidence indicate that LRRK2 toxicity is kinase-dependent [4–7], and the most common *LRRK2* mutation, *LRRK2-G2019S* shows increased kinase activity toward generic kinase substrates [4, 5, 8–11]. Therefore, to understand the physiological functions of LRRK2 as well as the mechanism by which mutations in LRRK2 contribute to PD pathogenesis, one of the greatest challenges in the field has been to identify authentic LRRK2 substrates that are associated with neurodegeneration. A previous study has suggested that ribosomal protein s15 is an endogenous substrate of LRRK2 which links LRRK2 toxicity to altered protein synthesis and neurodegeneration [12]. However, LRRK2 is thought to be a multivalent kinase and the precise mechanisms by which dysregulation of the kinase activity of LRRK2 causes neuronal toxicity are not fully understood.

The physiological functions of LRRK2 in neurons remain to be determined, but LRRK2 has been broadly implicated in membrane dynamics and vesicle trafficking. Previous studies have shown that LRRK2 localizes to membranous or vesicular structures, such as endosomes, lysosomes, multivesicular bodies, and autophagic vesicles [13–15], and increasing evidence suggests that LRRK2 might have a role in endocytosis [16, 17], endolysosomal sorting [18], retromer-mediated trafficking [19], and autophagy [13, 14, 20–23]. Rab GTPases, which comprise more than 60 members in the human genome, serve as multifaceted organizers in almost all membrane and vesicle trafficking processes [24, 25]. Since the identification of the first LRRK2-related Rab GTPase Rab5b, physical, genetic and functional interactions between LRRK2 and many Rab family members have been reported [18, 19, 26–28]. An unbiased search for interactors of LRRK2 and a brain transcriptomics approach concurred that LRRK2 interacts with Rab7L1 (also known as Rab29), and dysfunction of the LRRK2-Rab7L1 complex has been suggested to cause neurotoxicity by disrupting the endolysosomal and autophagic pathways [13, 19, 23, 27]. Large-scale phosphoproteomic screens using fibroblasts from knock-in mice that were genetically engineered to harbor either LRRK2-G2019S or inhibitor-resistant LRRK2-A2016T led to the identification of a single LRRK2 substrate, Rab10 [29]. Further analysis with selected Rab GTPases showed that a few more Rabs (Rab1b and Rab8a) were also directly phosphorylated by LRRK2 [29]. These findings are strongly suggestive of a physiological role for Rab phosphorylation by LRRK2. Thus, it is worthwhile to undertake a systematic inquiry on the interplay of LRRK2 and Rab family GTPases, and perhaps an equally important task is to interrogate the functional consequence of Rab phosphorylation by LRRK2, particularly with respect to neurodegeneration.

Here we performed an in vitro LRRK2 kinase assay with forty-five human Rab family proteins to screen Rab GTPases as potential substrates of LRRK2. Our screening revealed that Rab1a, 1b, 3, 8a, 8b, and 35 are directly phosphorylated by LRRK2, and we identified that Rab proteins are phosphorylated at a conserved threonine residue in the switch II region. By using the kinase-inactive LRRK2-D1994A and the pathogenic mutant LRRK2-G2019S along with phosphomutants of Rabs, we confirmed that a subset of Rab proteins, including Rab35, are authentic substrates of LRRK2 both in vitro and in cells. We also validated Rab phosphorylation at the endogenous level by using kinase inhibitors of LRRK2 and *Lrrk2* knockout mice. Moreover, substitution of the LRRK2 site in Rab1, 3, and 35 to either alanine or aspartate, but not the wild-types induced neurotoxicity, among which mutations in Rab35 caused the most severe phenotype. Furthermore, intracranial injection of adeno-associated viral vectors (AAVs) expressing phosphomutants of Rab35 but not the wild-type into the substantia nigra induced degeneration of dopaminergic neurons in vivo. To the best of our knowledge, this study is the first report on neurodegeneration of dopaminergic neurons caused by a direct substrate of LRRK2 in the mammalian brain.

## Methods

### Generation of recombinant Rab proteins

Entry clone cDNA for each Rab GTPases was subcloned into N-terminal GST tagged bacteria expression vector (pDEST15, Invitrogen, Carlsbad, CA, USA) using LR clonase (Invitrogen, Carlsbad, CA, USA) and the expression vectors generated were transformed into BL21-AI™ One Shot™ chemically competent *E.coli* (Invitrogen, Carlsbad, CA, USA). Protein expression was induced using 0.2% *w*/*v* L-arabinose (Sigma, St. Louis, MO, USA) for 1.5 h. Cells were pelleted (3000 *g* for 20 min) and then lysed with sonication in the presence of chilled lysis buffer (50 mM Tris-HCl (pH 7.5), 0.5% TX-100, 150 mM NaCl, 0.5 mM EGTA, 2 mM DTT and Complete Protease Inhibitor Mixture). Cell lysates were centrifuged at 15,000 *g* for 15 min, and the supernatant was incubated with GSH-sepharose beads (GE healthcare, Little Chalfont, UK) for overnight at 4°C. After incubation, the beads were washed with PBS containing 150 mM NaCl and 0.1% TX-100 three times. For the kinase assay, beads were stored in PBS containing 30% glycerol at − 80°C.

### In vitro LRRK2 kinase assays

Recombinant LRRK2 (Invitrogen, Carlsbad, CA, USA) and Rab proteins were incubated at 30°C for 30 min in kinase assay buffer (20 mM HEPES (pH 7.5), 5 mM EGTA, 20 mM β-glycerol phosphate, 20 mM MgCl_2_, 50 μM ATP (Sigma, St. Louis, MO, USA), and 0.5 μCi[γ-^32^P] ATP (PerkinElmer, Waltham, MA, USA)). The reaction was terminated by addition of Laemmili sample buffer, heated at 75°C for 10 min and then resolved by 10% SDS-PAGE. After fixation with 10% acetic acid and 40% methanol, gels were first stained in Coomassie brilliant blue (CBB), and the amount of each Rab GTPase was quantified by measuring the intensity of Rab protein bands from CBB staining using ImageJ. Then, the gels were exposed to an X-ray film, and the amount of ^32^P incorporation into the substrate (Rab protein of interest) and LRRK2 (autophosphorylation) was quantified by standard autoradiography using ImageQuant 6.0 software. The level of radiolabeled Rab proteins was normalized against both the amount of Rab GTPases (from CBB staining) and the level of LRRK2 autophosphorylation (from autoradiography) using the following equation:

$$ \frac{\left(\mathrm{amount}\ \mathrm{of}\ 32\mathrm{P}\ \mathrm{incorporated}\ \mathrm{into}\ \mathrm{substrate}\right)\times \mathrm{100,000}}{\ \left(\mathrm{amount}\ \mathrm{of}\ \mathrm{Rab}\ \mathrm{GTPase}\right)\times \left(\mathrm{amount}\ \mathrm{of}\ 32\mathrm{P}\ \mathrm{incorporated}\ \mathrm{into}\ \mathrm{LRRK}2\right)\ } $$

### Identification of phosphorylation sites by tandem mass spectrometry

Recombinant LRRK2 and Rab8a proteins were incubated at 30°C for 30 min in the kinase assay buffer (20 mM HEPES (pH 7.5), 5 mM EGTA, 20 mM β-glycerol phosphate, 2 mM ATP, and 20 mM MgCl_2_). The reaction was terminated by addition of Laemmili sample buffer, heated at 75°C for 10 min and then resolved by 10% SDS-PAGE. Gels were stained by CBB after fixation with 10% acetic acid and 40% methanol. Excised gel bands were destained with 50% ACN, shrunk with 100% ACN. Proteins in gels were then reduced for 30 min at 60 °C by adding 1 mM DTT and then alkylated for 30 min in dark place by the addition of 5.5 mM IAA solution. The samples were digested with sequence grade modified trypsin for overnight at 30 °C in 0.1 M NH_4_HCO_3_. About 0.1 μg of protease was used for one gel band. Peptides were extracted from the gel slices with 66% ACN and 5% FA. The combined supernatants and extracts were dried by speedvac, and phosphopeptides were enriched by TiO_2_ (GL science, Tokyo, Japan) microcolumns packed in GELoader tips. A small plug of C_8_ material was stamped out of a 3M Empore C_8_ extraction disk (3M, St. Paul, MN, USA) and placed at the end of GELoader tip. TiO_2_ beads were suspended in acetonitrile and packed on top of the C_8_ disk using a 1 mL disposable syringe. Peptide mixtures were diluted five times in loading buffer (1 M glycolic acid in 80% acetonitrile, 2-5% TFA) and loaded onto the TiO_2_ microcolumns. The column was washed with 10 μl of loading buffer followed by 40 μl of washing buffer (80% acetonitrile, 2% TFA). The samples were eluted using 20-40 μl of ammonia water (10 μl of 25% ammonia solution in 490 μl of water), pH 11. A small aliquot of each of the eluates were acidified with 4-8 μl of formic acid and purified using a SepPak microcolumn (Waters, Wexford, Ireland). The resultant samples were dried by speedvac. The resultant samples were dried by speedvac and stored at − 80 °C before analysis. The samples were redissolved in 20 μL of 5% FA and analyzed by on-line nanoflow LC-MS/MS. All nano-LC-MS/MS experiments were performed on an Ultimate 3000 system and nano-RSLC (Thermo Fisher Scientific, Bremen, Germany) connected to Mass Spectrometer with a nanoelectrospray ion source. The tryptic digested peptides were separated in a 15 cm analytical column (Zorbax 300SB C18, 0.075 mm × 100 mm, Agilent Technologies, Waldbronn, Germany) with a 90-min gradient from 5 to 60% acetonitrile in 0.1% formic acid. The effluent from nanoLC was directly electrosprayed into the mass spectrometer. The MS instruments (LTQ XL and LTQ-Orbitrap Elite, Thermo Fisher Scientific, Waltham, MA, USA) were operated in data-dependent mode to automatically switch between full-scan MS and MS/MS acquisition. In LTQ, MS spectra were acquired over the mass range of m/z 300–1600 Da in the ion trap at a resolution of 3000 and subsequently subjected to MS/MS with the five most intense peptide ions (charge states ≥2), which were sequentially isolated and fragmented in the linear ion trap by multistage activation (MSA; or pseudo-MS3). MS/MS spectra were identified by using the MASCOT v2.3 (Matrix Science, London, UK) against latest uniprot human or mouse database. To search the phosphorylation sites, we used the searching parameters as the following: maximum missing cleavage sites, 4; fixed modification, carbamidomethyl (C); variable modification, oxidation (M), biotin (K), biotin (N-term), phospho(Y), and phospho(S, T). The tolerances of MS spectra and MS/MS were used as 2 Da and 1 Da respectively. After positive identification by Mascot 2.3., we confirmed the results by manual inspection.

#### Rab plasmids and site-directed mutagenesis

Entry clones for Rab GTPases were subcloned into N-terminal V5- or GFP-tagged expression vectors (pcDNA™ 3.1/nV5-DEST, pcDNA™-pDEST53) (Thermo Fisher Scientific, Waltham, MA, USA) using LR clonase (Invitrogen, Carlsbad, CA, USA). Point mutant plasmids of Rab GTPases were generated using site-directed mutagenesis kit (Agilent Technologies, Santa Clara, CA, USA) according to the manufacturer’s protocol. Each entry clones were used as template and primer sequences were as follows:

Rab1a T75A (F: gaaagatttcgagcaatcacctcca-3′, R: tggaggtgattgctcgaaatctttc-3′),

Rab1a T75D (F: 5′-gaaagatttcgagacatcacctccagt-3′, R: 5′-actggaggtgatgtctcgaaatctttc-3′),

Rab3c T94A (F: 5′-gaaagatacagggctatcaccacag-3′, R: 5′-ctgtggtgatagccctgtatctttc-3′),

Rab3c T94D (F: 5′-gaaagatacagggatatcaccacagc-3′, R: 5′-gctgtggtgatatccctgtatctttc-3′),

Rab8a T72A (F: 5′-gaacggtttcgggcgatcacaacgg-3′, R: 5′-ccgttgtgatcgcccgaaaccgttc-3′),

Rab8a R72D (F: 5′-gaacggtttcgggacatcacaacggcc-3′, R: 5′-ggccgttgtgatgtcccgaaaccgttc-3′),

Rab35 T72A (F: 5′-gagcgcttccgcgccatcacctcca-3′, R: 5′-tggaggtgatggcgcggaagcgctc-3′),

Rab35 T72D (F: 5′-gagcgcttccgcgacatcacctccac-3′, R: 5′-gtggaggtgatgtcgcggaagcgctc-3′),

Rab1a Q70L (F: 5′-cacagcaggcctggaaagatttc-3′, R: 5′-gaaatctttccaggcctgctgtg-3′),

Rab1a S25 N (F: 5′-gggttggaaagaattgccttcttc-3′, R: 5′-gaagaaggcaattctttccaaccc-3′),

Rab3c Q89L (F: 5′-cacagcaggcctggaaagataca-3′, R: 5′-tgtatctttccaggcctgctgtg-3′),

Rab3c T44 N (F: 5′-tgtggggaaaaattcttttctatt-3′, R: 5′-aatagaaaagaatttttccccaca-3′),

Rab8a Q67L (F: 5′-cacagccggtctggaacggtttc-3′, R: 5′-gaaaccgttccagaccggctgtg-3′),

Rab8a T22 N (F: 5′-ggtggggaagaactgtgtcctgt-3′, R: 5′-acaggacacagttcttccccacc-3′),

Rab35 Q67L (F: 5′-cacagcggggctggagcgcttcc-3′, R: 5′-ggaagcgctccagccccgctgtg-3′),

Rab35 S22 N (F: 5′-tgtgggcaagaacagtttactgt-3′, R: 5′-acagtaaactgttcttgcccaca-3′).

### Generation of phospho-Rab antibodies

Polyclonal antibodies against Rab1a phospho-T75 were generated by injection of KLH-conjugated phospho-peptide TAGQERFRpTITSSYYRG into rabbits. Antibodies were purified from crude sera by using a sulfo-linked immobilized peptide affinity column (Thermo Fisher Scientific, Waltham, MA, USA) containing Rab1a phospho-peptide. The eluent was then applied to a sulfo-linked immobilized peptide affinity column containing non-phosphorylated Rab1 peptide. We obtained purified phospho-antibody from the flow-through.

### GTP binding assay

Myc-tagged LRRK2s (wild-type, G2019S or D1994A) and/or V5-tagged Rabs (wild-type or their phospho-mutants of Rab1a, 3c, 8a, 35) were transfected with Lipofectamine 2000 (Invitrogen, Carlsbad, CA, USA) into HEK-293 cells. Cells were lysed with GTP-binding buffer (50 mM Tris-HCl (pH 7.5), 0.5% Triton-X 100, 150 mM NaCl, 5 mM MgCl2, 5 mM EGTA, Protease Inhibitor Mixture (50 μM aprotinin, 50 μM leupeptin, and 50 μM pepstatin), and 0.2 mM PMSF) and centrifuged (15,000 *g* for 15 min). Supernatants were incubated with immobilized γ-amino-hexyl-GTP agarose (Jena Bioscience, Jena, Germany) for 12 h at 4°C and then the beads were washed with GTP-binding buffer containing 0.1% TX-100. GTP-bound Rabs were eluted by Laemmili sample buffer and subjected to SDS-PAGE for western blot analysis.

### LRRK2 and Rab co-immunoprecipitation

Myc-tagged LRRK2 and V5-tagged Rabs (Rab1a, 3c, 8a, or 35) were co-transfected with Lipofectamine 2000 into HEK-293 cells. Cells were lysed in lysis buffer (50 mM Tris-HCl (pH 7.5), 0.5% TX-100, 150 mM NaCl, 0.5 mM EGTA, protease inhibitor mixture (50 μM aprotinin, 50 μM leupeptin, and 50 μM pepstatin, and 0.2 mM PMSF) and then centrifuged (15,000 *g* for 15 min). Supernatants were incubated with anti-V5 or Myc antibodies coupled to protein-G-agarose for overnight at 4°C and then washed with PBS containing 150 mM NaCl and 0.1% TX-100 for three times. The beads bound to proteins were eluted by Laemmili sample buffer and subjected to SDS-PAGE for western blot analysis.

#### Preparation of tissues for immunoblot

Whole brains from *Lrrk2* knock-out or wild-type mice (Fig. 3e), ventral midbrain regions from AAV-injected mouse (Additional file 2: Figure S2), or various brain regions (olfactory bulb, cortex, hippocampus, striatum, ventral midbrain, cerebellum, and brain stem) from C57/BL6 mice (Additional file 4: Figure S4) were homogenized in lysis buffer (50 mM Tris-HCl (pH 7.5), 0.5% TX-100, 150 mM NaCl, 0.5 mM EGTA, Protease Inhibitor Mixture (50 μM aprotinin, 50 μM leupeptin, and 50 μM pepstatin), and 0.2 mM PMSF) with dounce tissue grinder. The homogenates were incubated for 30 min on ice and then centrifuged for 15 min at 4 °C. Protein levels of the supernatants were quantified with BCA kit (Thermo Fisher Scientific, Waltham, MA, USA) and western blot analysis was performed with anti-Rab35 (1:1000, 11,329-2-AP, Proteintech, Rosemont, IL, USA), anti-TH (1:2000, P40101, Pel Freez, Rogers, AK, USA), and anti-actin antibodies.

### Animals

All experiments involving animals were performed in accordance with the guidelines of the Institutional Animal Care and Use Committee of the Korea Institute of Science and Technology and Kyung Hee University. Pregnant ICR mice and C57BL6 were purchased from DBL (Eumseong, South Korea) and housed in the Korea Institute of Science and Technology Animal Facility. C57BL6 mouse (7-weeks old) were acclimatized for one week under conditions of controlled temperature (22 ± 2°C), constant humidity, and a 12-h light/dark cycle, and food and water were made available ad libitum. All surgical procedures were conducted according to the animal welfare guidelines approved by the Kyung Hee University Institutional Animal Care and Use Committee (KHUASP(SE)-16-074).

### Primary cell culture and plasmid transfection

Cortical neurons were prepared from embryonic day 15.5 mice as described elsewhere. Cortices were dissected in HBSS (Gibco, Waltham, MA, USA), followed by digestion in papain (20 U ml^− 1^, Worthington, Lakewood, NJ, USA) diluted in HBSS containing DNase (10 U ml^− 1^, Sigma, St. Louis, MO, USA) for 40 min at 37 °C. Enzyme-digested cortices were washed three times with MEM (Cellgro, Manassas, VA, USA) containing 10% heat inactivated fetal bovine serum (Hyclone, Little Chalfont, UK) and dissociated in culture medium. Dissociated neurons were then centrifuged to remove the supernatant, and cells were plated in 24-well dishes with 12 mm glass coverslips (5 × 10^4^ cells per well) coated with 100 μg ml^− 1^ poly-D-lysine (Sigma, Carlsbad, CA, USA). Cells were cultured in Neurobasal A (Gibco, Waltham, MA, USA) medium containing 1% Glutamax (Thermo Fisher Scientific, Waltham, MA, USA) and 2% B27 (Thermo Fisher Scientific, Waltham, MA, USA) supplements, and 1% penicillin/streptomycin. At one day prior to transfection, media was replaced to Neurobasal A (Gibco, Waltham, MA, USA) medium containing 1% Glutamax and 2% B27 (Thermo Fisher Scientific, Waltham, MA, USA) supplements to remove antibiotics. At DIV 7, neurons were transfected with plasmid DNA (Myc-tagged LRRK2, GFP-conjugated Rab, or EGFP as a control) using Lipofectamine 2000 in Opti-MEM reduced serum medium. Neurons were fixed at the indicated time points and immunostained with either anti-myc (for cells transfected with LRRK2 constructs) or anti-GFP antibodies (for cells transfected with Rab constructs) to identify transfected cells.

### Immunostaining and fluorescence microscopy

Neurons were fixed in a solution containing pre-warmed 4% paraformaldehyde (PFA), 0.15% glutaraldehyde, and 0.2% Triton X-100 dissolved in PBS (37 °C, 20 min). Fixed neurons were blocked in a blocking solution (2% BSA, 0.2% Triton X-100 in PBS). Primary (GFP, PA1-9533, Thermo Fisher Scientific, Waltham, MA, USA, 1:500; Flag, TA100023, OriGene, Rockville, MD, USA) antibodies were diluted in the blocking solution and secondary antibodies (Alexa fluor 488, A11039, Invitrogen, Carlsbad, CA, USA) in PBS. All secondary antibodies (1:400-500) were incubated for 1 h at room temperature. After extensive rinsing with PBS, coverslips were mounted onto glass slides for observation. All coverslips in any one experiment were fixed and processed together. Neurons were viewed under an inverted microscope (Axio Observer Z1, Carl Zeiss MicroImging, Inc., Jena, Germany) equipped with epifluorescence optics. Images were captured with a CCD camera controlled by the ZEN software (Carl Zeiss MicroImaging, Inc., Jena, Germany). A 10× objective (0.45 NA) was used to record whole neurons. Images were cropped to more clearly show neuronal cell morphology and improve conciseness of the presentation.

### LRRK2 toxicity assays

Transfected neurons were identified either by myc (for wile-type and mutants of LRRK2) or GFP immunostaining (for wild-types and mutants Rab proteins). Cells included in the analysis were also positive for TuJ1 (Additional file 1: Figure S1). For Fig. 5, neurons expressing LRRK2 or Rab of interest (identified by either anti-myc or anti-GFP antibodies) that extended at least one neurite longer than twice the cell body diameter were photographed, and the length of the longest neurite in each neuron was manually traced using the measure/curve application of Zen blue software (Carl Zeiss Microimaging, Inc.). Neurite length was analyzed from three (for 1 and 7 day point) or five (for 2 day point) independent experiments, and in any one experiment, at least 20 transfected neurons per construct were measured. For Fig. 6, neurons were also subjected to TUNEL staining (C10246, Invitrogen, Carlsbad, CA, USA) in accordance with the manufacturer’s protocol. For quantification, images were taken from 15 to 20 (10X) randomly selected fields per each condition using a Zeiss Axiocam fluorescent microscope with Axiovision 6.0 software. The percentage of neurons expressing the plasmid of interest exhibiting TUNEL-positive nuclei was quantified. For Fig. 6a and b, six independent experiments were performed, and in any one experiment at least 40 transfected neurons per construct were counted.

#### Generation of AAV constructs and virus particles

A plasmid for AAV vectors with the human synapsin 1 gene promoter (hSyn) was used for the expression of eGFP alone, or eGFP and hRab35 WT, hRab35 T72A, or hRab35 T72D via P2A peptide-mediated co-expression system. AAV vectors were produced according to the protocol provided by the Salk Institute viral vector core facility (http://vectorcore.salk.edu/index.php) with slight modifications. In brief, HEK-293 cells were co-transfected with a mixture of three plasmids, one AAV plasmid, the pHelper plasmid (Agilent Technologies, Santa Clara, CA, USA), and the serotype 1 plasmid (Vector Core of the University of Pennsylvania), using calcium phosphate. At 72 h after transfection, cells were harvested and lysed by sonication. Then, AAV vectors were collected from the cell lysate by gradient ultracentrifugation using a Beckman NVT90 rotor at 183000 *g* for 47 min, dialyzed in PBS with D-sorbitol, concentrated by centrifugal filter devices (Millipore, Billerica, MA, USA), and stored at -80 °C. The AAV titer was estimated by quantitative PCR of DNase-I-treated AAV (2-4 × 10^13^ vector genome copies/ml).

#### Virus injection and stereological assessment

Stereotaxic injections were performed on 8-week old mice. AAV-hSyn-P2A-eGFP, AAV-hSyn-hRab35 WT-P2A-eGFP, AAV-hSyn-hRab35 T72A-P2A-eGFP, or AAV-hSyn-hRab35 T72D-P2A-eGFP was injected unilaterally into the substantia nigra (A.P. -3.1. M.L. -1.4, D.V. -4.5) (0.3 μl per site) at a rate of 0.3 μl per 10 min with a 10 μl Hamilton syringe driven by a syringe pump. For each condition, five animals were injected. Three weeks after virus administration, the mice were anesthetized with pentobarbital (50 mg kg^− 1^, intraperitoneal injection) and perfused with PBS followed by 4% PFA (*w*/*v* in PBS). Brains were post-fixed with 4% PFA overnight and cryoprotected in 30% sucrose (w/v in PBS) overnight. Forty μm coronal sections were made throughout the brain including striatum or substantia nigra, and every fourth section was utilized for analysis. Sections were stained with rabbit polyclonal anti-tyrosine hydroxylase (TH) (1:2000, P40101, Pel Freez, Rogers, AK, USA) antibodies and visualized with biotinylated goat anti-rabbit IgG, followed by streptavidin-conjugated horseradish peroxidase (HRP) (Vectastain ABC kit, Vector Laboratories, Burlingame, CA, USA). Positive immunostaining was visualized with 3,3′-diaminobenzidine (DAB, Sigma, St. Louis, MO, USA) after reaction with hydrogen peroxide (DAB kit, Vector Laboratories). For Nissl staining, sections were stained with 0.5% cresyl violet acetate (Sigma, St. Louis, MO, USA). Sections were sequentially dehydrated with in 25, 50, 75, 90, and 100% ethanol and cleared in xylene. Total numbers of TH-or Nissl-positive neurons in the substantia nigra pars compacta were counted using the optical fractionator probe of Stereo Investigator software (MicroBrightfield, Williston, VT, USA). Experimenters were blinded to the treatment during stereological counting. For immunohistochemistry, fixed brain sections including the substantia nigra were immunostained with anti-GFP (1:1000, A11120, Life Technologies, Carlsbad, CA, USA) and anti-TH (1:1000, ab112, Abcam, Cambridge, UK) antibodies overnight at 4 °C. Secondary antibodies, Alexa 488 (1:1000, A11029, Life Technologies, and Carlsbad, CA, USA) and Alexa 568 (1:1000, A10042, Life Technologies, Carlsbad, CA, USA) were incubated for 1 h at room temperature. After extensive rinsing with PBS, sections were mounted onto glass slides for observation. Sections were viewed under an inverted microscope (LSM 700 confocal laser scanning microscope (Carl Zeiss MicroImging, Inc., Jena, Germany)).

#### Fractionation of membrane

Membrane fraction was prepared, as described previously [30]. V5-tagged Rab35 wild-type, T75A, or T75D mutants were transfected with Lipofectamine 2000 into HEK-293. Cells were washed once with PBS and scraped in lysis buffer containing 0.25 M sucrose, 1 mM EDTA, 10 mM Hepes-NaOH (pH 7.5), 1 mM MgCl_2_ and protease inhibitors. Cell lysates were passed through a 27 gauge needle 10 times and centrifuged at 1000 g for 15 min at 4 °C. Protein levels were quantified using the BCA kit (Thermo Fisher Scientific, Waltham, MA, USA). Equal amount of protein from each sample was loaded to centrifuge tubes (Beckman Coulter, Indianapolis, IN, USA) and centrifuged using an SW 32 Ti swinging bucket rotor (Beckman Coulter, Indianapolis, IN, USA) for 1 h at 100,000 g at 4 °C. The pellets were rinsed with lysis buffer and centrifuged for 1 h at 100,000 g at 4 °C. Pellets were resuspended in Laemmini sample buffer and subjected to SDS-PAGE. Western blot analysis was performed with anti-V5, anti-caveolin-1 (1:1000, 3267, Cell Signaling, Danvers, MA, USA), anti-EEA1 (1:1000, 3288, Cell Signaling, Danvers, MA, USA), anti-GOPC (1:1000, 8576, Cell Signaling, Danvers, MA, USA), and anti-Hsp90 (1:1000, ab13492, Abcam, Cambridge, UK) antibodies.

### Statistics

All data presented in this study were either averages or representative data from at least three independent experiments, except the mass spectrometry data. The number of independent experiments and sample size are indicated under each section of the Methods and in the figure legends. Statistical analyses were conducted using the software Graph-Pad Prism (GraphPad Software 7, Inc.). Prior to determining statistical significance, Shapiro-Wilk test was performed to assess normality. For Figs. 4, 5, and 6, one-way ANOVA with Dunnett’s post hoc test was performed, and for Fig. 7, one-way ANOVA was followed by Tukey’s post hoc test. Data were expressed as mean ± SD. *, **, *** and **** in the figures denote *p* < 0.05, 0.01, 0.001, and 0.0001, respectively.

## Results

### A targeted screen for LRRK2 substrates using recombinant Rab GTPases

**Fig. 1:**
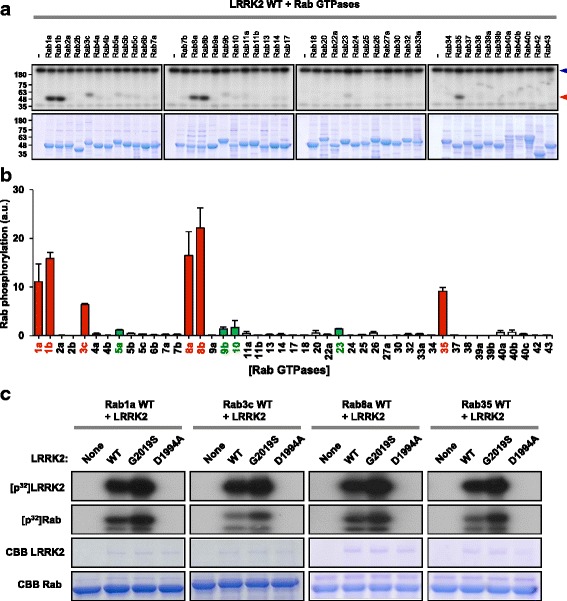
Phosphorylation of Rab GTPases by LRRK2. **a** Representative phosphoimages and commassie brilliant blue (CBB)-stained gel images from the in vitro LRRK2 kinase assays with forty-five Rab GTPases. LRRK2 autophosphorylation and Rab phosphorylation are indicated by blue and red arrowhead, respectively. **b** The level of Rab phosphorylation was normalized against both the protein level of each Rab from CBB staining and the level of LRRK2 autophosphorylation. Extent of phosphorylation is color-coded: potent phosphorylation in red and marginal in green. Data are mean ± SD (*n* = 3). **c** Representative phosphoimages and CBB-stained gel images from LRRK2 in vitro kinase assays with Rab1a, 3c, 8a, and 35

To investigate which of the Rab GTPases family are direct substrates of LRRK2, we generated GST-fusion proteins for forty-five human Rab GTPases by Gateway cloning and performed in vitro kinase assays with wild-type LRRK2 (Fig. 1a). In any one independent experiment, we included all 45 Rab GTPases and performed the in vitro kinase assays side by side to compare phosphorylation efficiencies. The extent of phosphorylation on each Rab was calculated by normalizing Rab phosphorylation against both the amount of Rab GTPases determined by Coomassie brilliant blue staining and the level of LRRK2 autophosphorylation determined by standard autoradiography as described in Methods. In vitro kinase assay revealed that LRRK2 directly phosphorylated a subset of Rab GTPases to varying degrees. The most prominent substrates that were strongly phosphorylated by LRRK2 included Rab1a, 1b, 3c, 8a, 8b, and 35. We found that Rab5a, 9b, 10, and 23 were also phosphorylated but to a much lower extent (Fig. 1a and b). Interestingly, the level of Rab phosphorylation was well correlated with their sequence homology. Rab GTPase family can be classified into ten subfamilies based on their distinct subfamily-specific sequence motifs [31]. Rab1a, 1b, and 35 are classified as Rab1 subfamily, and Rab8 subfamily is localized close to Rab1 subfamily in the phylogenic tree [31]. Rab3 subfamily is also in a relatively proximate position with Rab1 and 8 families compared to other subfamilies [32]. With Rab1a, 3c, 8a, and 35, which showed discernable phosphorylation in the initial screening, we performed additional in vitro kinase assays in the presence of kinase-dead LRRK2-D1994A or pathogenic LRRK2-G2019S that has increased kinase activity (Fig. 1c). As expected, none of the Rabs were phosphorylated by LRRK2-D1994A, while LRRK2-G2019S induced stronger phosphorylation compared to wild-type LRRK2.

### Identification of LRRK2 phosphorylation sites in Rab GTPases

**Fig. 2:**
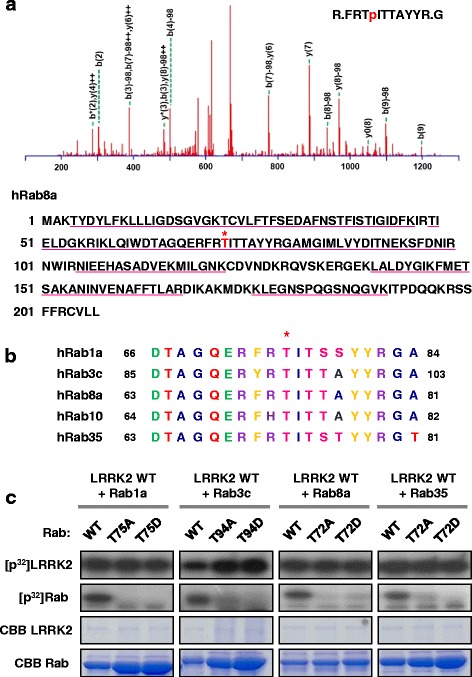
Identification of Rab phosphorylation site. **a** (*Top*) Tandem mass spectrometry (MS/MS) spectrum of Rab8a phosphorylated by LRRK2 indicates T72 as a phosphorylation site. (*Bottom*) Underlined text indicates sequence coverage by MS/MS. Phosphorylation site is indicated in red with an asterisk. **b** Local amino acid sequence alignment surrounding the LRRK2 phosphorylation site in Rab GTPases. Phosphorylation site is indicated with an asterisk. Amino acids are color-coded: aliphatic amino acids in blue, polar in red, acidic in green, basic in purple, and aromatic in yellow. **c** Validation of phosphorylation site identified by MS/MS. Representative phosphoimages and CBB-stained gel images from LRRK2 in vitro kinase assays performed in the presence of phosphodeficient or phosphomimetic mutant of each Rab (Rab1a T75A or T75D; Rab3c T94A or T94D, Rab8a T72A or T72D; Rab35 T72A or T72D)

To identify the phosphorylation site on Rab GTPases, tandem mass spectrometry (MS/MS) analysis was performed. Incubation of Rab8a with wild-type LRRK2, followed by MS/MS analysis revealed T72 as a potential phosphorylation site (Fig. 2a). This threonine residue, which is located in the switch II region of Rab8a, is highly conserved among multiple Rab GTPases and is found in all of the Rabs that we identified as LRRK2 substrates in the in vitro kinase assay (Fig. 2b). Through unbiased screening of an oriented peptide library, a previous study suggested F/Y-X-T-X-R (underlined T is the phosphorylation site) sequence as a LRRK2 phosphorylation motif, which partially overlaps with the presumed phosphorylation site in Rab8a. To confirm that T72 in Rab8a was the LRRK2 site, we substituted the threonine residue to alanine (Rab8a-T72A) and performed in vitro LRRK kinase assay (Fig. 2c). LRRK2 failed to phosphorylate Rab8a-T72A, verifying that the predicted site was indeed the primary phosphorylation site. Next, to examine if LRRK2 also phosphorylated the equivalent threonine residues in other Rab GTPases, we mutated the corresponding threonine in each Rab and performed in vitro LRRK2 kinase assays (Fig. 2c). Substitution of the putative LRRK2 phosphorylation site to either alanine (TA) or aspartate residue (TD) in Rab1a, 3c, 8a and 35 abolished phosphorylation in all Rabs tested, verifying that the conserved threonine residues were the major LRRK2 phosphorylation sites.

### Phosphorylation of Rab by LRRK2 in cells

**Fig. 3:**
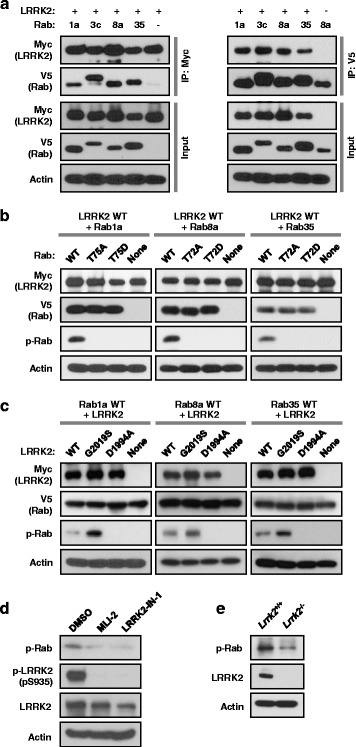
LRRK2 and Rab interaction and Rab phosphorylation by LRRK2 in cells. **a** Co-immunoprecipitation of LRRK2 and Rab proteins. Human embryo kidney (HEK)-293 cells were co-transfected with myc-tagged LRRK and V5-tagged Rab1a, 3c, 8a, or 35, and immunoprecipitated using anti-myc or V5 antibodies, followed by western blot analysis. (b-c) Verification of Rab phosphorylation by LRRK2 in HEK-293 cells. Expression and phosphorylation of Rab and/or LRRK2 were monitored by western blot analysis, and actin level was monitored as a loading control. Rab phosphorylation was examined by phospho-site-specific Rab antibodies (p-Rab) generated against a phosphopeptide corresponding to the LRRK2 site in Rab1a, as described in Methods. For **b**, cells were transfected either with LRRK2 wild-type (WT) alone (depicted as “None” in each set) or along with WT or phosphomutants (TA or TD) of Rab1a, 8a, or 35. For **c**, cells were transfected either with Rab1a, 8a, or 35 (all WTs) alone (depicted as “None” in each set) or along with WT or mutants (G2019S or D1994A) of LRRK2. **d** NIH-3T3 cells were treated with MLi-2 (1 μM), LRRK2-IN-1 (1 μM), or vehicle control, and phosphorylation of endogenous Rab and endogenous LRRK2 was monitored by western blot analysis using anti-p-Rab and anti-pLRRK2 (phospho-serine 935) antibodies. **e** Phosphorylation of endogenous Rab in WT and *Lrrk2* knockout mice. Brain lysates from WT and *Lrrk2* knockout mice were subjected to western blot analysis with the indicated antibodies. All results shown are representative blots from three independent experiments

To extend the findings from the in vitro kinase assays, we first investigated whether LRRK2 interacts with Rabs in cells. For co-immunoprecipitation analysis, we ectopically expressed myc-tagged LRRK2 along with the V5-tagged Rab of interest in human embryonic kidney (HEK)-293 cells. Immunoprecipitation of cell lysates with anti-myc antibodies, followed by immunoblotting with anti-V5 antibodies revealed that LRRK2 was bound to Rab1a, 3c, 8a, and 35 (Fig. 3a). Reciprocal co-immunoprecipitation experiments using antibodies against V5 for immunoprecipitation, followed by immunoblotting with anti-myc antibodies confirmed that all of the Rabs tested physically interacted with LRRK2.

To validate the in vitro phosphorylation of Rabs in cells, we raised phospho-specific antibodies against a phosphopeptide TAGQERFRpTITSSYYRG, corresponding to the highly conserved sequence surrounding the LRRK2 site in Rab1a (see Fig. 2b). In HEK-293 cells, phosphorylation of Rab1a, 8a, and 35 was readily detected when we co-expressed both LRRK2 and the Rab of interest, but not when we overexpressed either LRRK2 or Rab (Fig. 3b and c). Despite the observation that LRRK2 directly phosphorylated Rab3c in vitro, we failed to detect phosphorylation of Rab3c from cell lysates (data not shown), which might be because of a slightly different amino acid sequence close to the LRRK2 site (note the tyrosine residue in Rab3c instead of phenylalanine in all other Rabs at position − 2 with respect to the LRRK2 site). Interestingly, when we co-transfected Rab10 and LRRK2, the extent of phosphorylation of Rab10 was comparable to those of Rab1a, 8a, and 35 (data not shown), despite the observation that Rab10 was not a prominent substrate of LRRK2 in the in vitro kinase assay (see Fig. 1a). When we co-transfected HEK-293 cells with phosphomutants (phosphomimetic TD or phosphodeficient TA mutant) of the Rab of interest along with wild-type LRRK2, we could not detect any phosphorylation (Fig. 3b), confirming that the conserved threonine sites in Rab GTPases were the targets of LRRK2 in cells. Furthermore, the extent of phosphorylation of Rab1a, 8a, and 35 was enhanced by LRRK2-G2019S compared to wild-type LRRK2, while none of the Rabs were phosphorylated by kinase-dead LRRK2-D1994A (Fig. 3c). Together, these results confirm the specificities of the phospho-Rab antibodies that we generated, and more importantly, provide unambiguous evidence that Rab1a, 8a, and 35 are phosphorylated by LRRK2 both in vitro and in cells.

To examine phosphorylation of Rab proteins at the endogenous level, we first examined the effects of LRRK2 inhibitors in NIH-3T3 fibroblast cells, which express endogenous LRRK2. More specifically, we treated NIH-3T3 cells with two different inhibitors of LRRK2, either LRRK2-IN-1 (1 μM) or MLi-2 (1 μM), and examined the changes in Rab phosphorylation using the aforementioned phospho-specific Rab antibodies that we generated (see Fig. 3b, c). Inhibition of endogenous LRRK2 by the two inhibitors was confirmed by immunoblotting with phospho-specific LRRK2 (phospho-serine 935) antibodies, and importantly, we confirmed that LRRK2-IN-1 or MLi-2 markedly reduced phosphorylation of Rab (Fig. 3d). Furthermore, we also confirmed that the level of phospho-Rab was substantially diminished in brain lysates from *Lrrk2* knock-out mice compared to wild-type (Fig. 3e). Collectively, these results provide strong support for the notion that endogenous Rab proteins are phosphorylated by endogenous LRRK2.

### Phosphorylation of Rab by LRRK2 affects GTP-binding

**Fig. 4:**
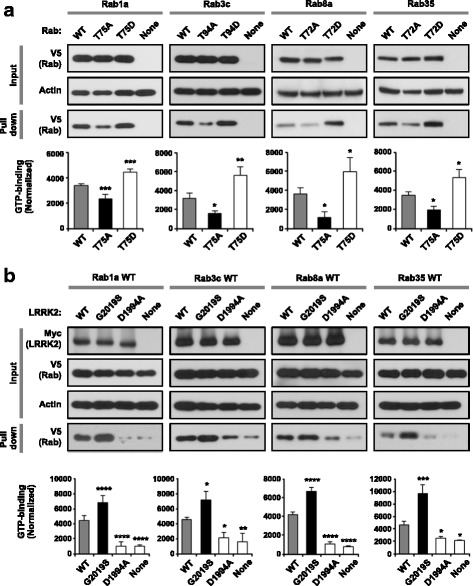
Regulation of Rab GTP binding ability by LRRK2-induced phosphorylation. **a**, **b** GTP-binding assay for Rab1a, 3c, 8a, and 35. In **a**, HEK-293 cells were transfected with either wild-type (WT) or phosphomutants (TA or RD) of Rab1a, 3c, 8a, or 35. In **b**, HEK-293 cells were transfected with Rab1a, 3c, 8a, or 35 (all WTs) alone (depicted as “None” in each set) or together with WT or mutants (G2019S or D1994A) of LRRK2. In **a** and **b**, cell lysates were incubated with γ-amino-hexyl-GTP agarose and GTP-bound Rabs were subjected to SDS-PAGE for western blot analysis. Shown are representative immunoblot images (*top*) and quantification of GTP-binding Rab levels (*bottom*) from three independent experiments. Data are mean ± SD (*n* = 3). * *p* < 0.05, ** *p* < 0.01, *** *p* < 0.001, and **** *p* < 0.0001; one-way ANOVA followed by Dunnett’s multiple comparison post hoc test

To elucidate the functional consequence of LRRK2-induced Rab phosphorylation, we investigated the changes in GTP-binding ability of Rab GTPases after LRRK2 phosphorylation. Rab GTPases cycle between active (GTP-bound) and inactive (GDP-bound) states, which is controlled by three groups of regulatory proteins: GTPase-activating proteins (GAPs), guanine nucleotide exchange factor (GEFs), and guanine nucleotide-dissociation inhibitors (GDIs). The conserved threonine residue (LRRK2 site) resides in the switch II region, which undergoes a major conformational transition between GDP- and GTP-bound states and coordinates the association with specific regulatory molecules [33]. We tested whether the mutation of the threonine residue affected the GTP hydrolysis property of Rab GTPases by using hydrolyzable GTP agarose, reflective of GTPase activity at the time of precipitation. For all Rabs tested, GTP-binding was reduced by substituting the threonine residue to alanine but increased by replacing it to aspartate (Fig. 4a). When LRRK2-G2019S was co-expressed with the Rab of interest, GTP-binding was increased compared to wild-type LRRK2, whereas overexpression of LRRK2-D1994A had little effect on GTP-binding (Fig. 4b). These results suggest that phosphorylation of Rab proteins by LRRK2 kinase regulates the GDP/GTP exchange and that hyperactivation of LRRK2 kinase increases GTP-bound Rabs in cells.

### Mutations in the LRRK2 site in Rab GTPases cause neurodegeneration in cortical neurons

**Fig. 5:**
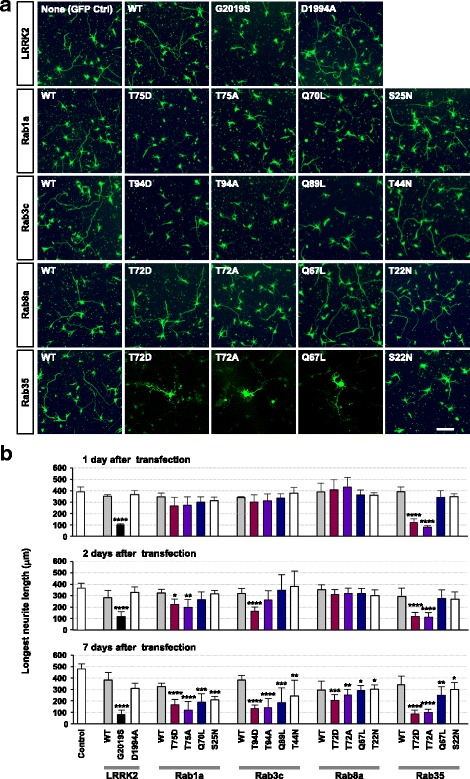
Phosphomutants of Rab induce neurotoxicity in primary cortical neurons. **a**, **b** Embryonic day 15.5 cortical neurons were transfected with wild-type (WT) or mutants of myc-tagged LRRK2 or GFP-conjugated Rab (Rab1a, 3c, 8a, or 35) at DIV 7. Neurons were fixed at the indicated time points after transfection and immunostained with either anti-myc (for cells transfected with LRRK2 constructs) or anti-GFP (for cells transfected with Rab constructs) antibodies to identify transfected cells. Representative images (**a**) and quantification of neurite length (**b**) are shown. Data represent mean ± SD from three (**a**) or five (**b**) independent experiments, and in any one experiment, at least 20 neurons per construct were analyzed

**Fig. 6:**
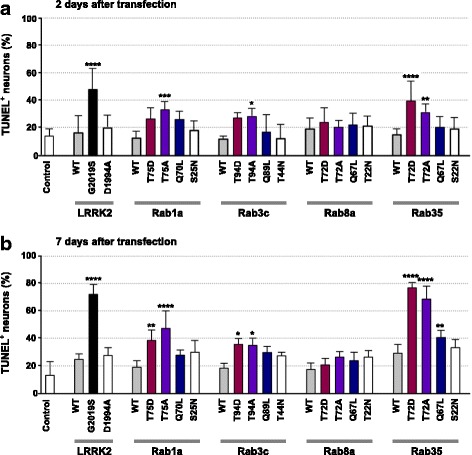
Neurotoxicity induced by LRRK2 substrate Rab GTPases in primary cortical neurons. **a**, **b** Embryonic day 15.5 cortical neurons were transfected at DIV 7 as in Fig. 5 and fixed at 2 (**a**) or 7 days (**b**) after transfection. Neurons were immunostained with either anti-myc (for cells transfected with LRRK2 constructs) or anti-GFP (for cells transfected with Rab constructs) antibodies to identify transfected cells. Neurons were also immunostained for TUNEL, and TUNEL-positive neurons were counted and presented as percentage of total neurons expressing the construct of interest. Data are mean ± SD from six independent experiments, and in any one experiment, at least 40 neurons per construct were analyzed. * *p* < 0.05, ** *p* < 0.01, *** *p* < 0.001, and **** *p* < 0.0001; one-way ANOVA followed by Dunnett’s multiple comparison post hoc test

Our results described above and those reported by others [29] suggest that Rab GTPases are authentic substrates of LRRK2. However, physiological consequence of such phosphorylation in neurons, and more importantly, its relevance to neurodegeneration remains unknown. In neurons, shortening or fragmentation of neurites has often been recognized as the hallmark of neurotoxicity, and pathogenic LRRK2 mutants induce neurite shortening, which ultimately leads to cell death [9]. To evaluate the effects of phosphorylation of Rab proteins by LRRK2 on neurotoxicity, cortical neurons were transfected with wild-type or mutants of LRRK2 or Rab GTPases, and neurotoxicity was examined by quantification of neurite length (Fig. 5) and TUNEL assay (Fig. 6). To avoid the possible effects on neurite development, we transfected the neurons with the plasmids at DIV 7, when neurons were fully polarized and matured. Consistent with a previous report [9], overexpression of LRRK2-G2019S but not the wild-type led to a dramatic shortening of neurites (Fig. 5) and ultimately to cell death (see Fig. 6). In the case of Rab1a, 3c, and 35, phosphomutants induced neurotoxicity, which was most severe in neurons expressing Rab35 mutants (Fig. 5b). At one day after transfection, neurotoxicity induced by phosphomutants of Rab35 was already evident, and at two days after transfection, phosphomutants of Rab1a and 3c also started to induce neurite shortening. By seven days after transfection, all of the mutants of Rab but none of the wild-types tested caused neurite shortening. Interestingly, phosphomutants of Rab8a did not exert overt neurotoxicity at early time points (Fig. 5b) despite that the extent of Rab8a phosphorylation induced by LRRK2 in the in vitro kinase assay was comparable to other Rabs (see Fig. 1a).

In the TUNEL assay, at 2 days after transfection, TA mutants of Rab1a, 3c, and 35, and the TD mutant of Rab35 caused neurotoxicity, which became much more prominent at 7 days after transfection (Fig. 6). By that time, TD mutants of Rab1a, 3c, and 35 and the QL mutant of Rab35 also resulted in neurodegeneration. Notably, neurodegeneration induced by phosphomutants of Rab35 was most severe compared to mutants of other Rabs, consistent with the effects on neurite shortening.

### Dysregulated phosphorylation of Rab35 causes degeneration of dopaminergic neurons in vivo

**Fig. 7:**
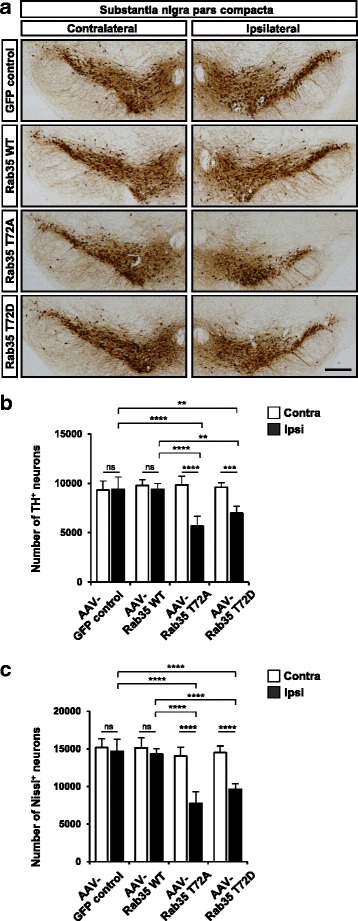
Phosphomutants of Rab35 induce degeneration of dopaminergic neurons in the substantia nigra. **a** Tyrosine hydroxylase (TH) immunostaining in the substantia nigra at 3 weeks after adeno-associated virus (AAV)-mediated delivery of wild-type (WT) or phosphomutants (T72A or T72D) of Rab35. AAV-eGFP was injected as a control. **b**, **c** Quantification of TH- (**b**) and Nissl-positive (**c**) neurons in the substantia nigra in the contralateral and ipsilateral side. Data are mean ± SD (*n* = 5). * *p* < 0.05, ** *p* < 0.01, *** *p* < 0.001, and **** *p* < 0.0001, n.s. statistically not significant; one-way ANOVA followed by Tukey’s post hoc analysis. Scale bar, 1 mm

To test whether dysregulation of the LRRK2 site in Rab induces neurodegeneration in vivo, we selected Rab35 whose mutation resulted in the most potent neurodegeneration in culture. We constructed adeno-associated viral (AAV) vectors in which synapsin promoter derives the expression of wild-type or mutants (T72A or T72D) of Rab35. To facilitate visualization of Rab35-expressing cells, Rab proteins were linked to enhanced green fluorescence protein (eGFP) via a self-cleaving P2A peptide. AAV-hSyn-Rab35 WT-P2A-GFP (AAV-Rab35 WT), AAV-hSyn-Rab35 T72A-P2A-GFP (AAV-Rab35 T72A), AAV-hSyn-Rab35 T72D-P2A-GFP (AAV-Rab35 T72D) or AAV-hSyn-P2A-GFP as a control (AAV-control) was stereotaxically injected into the substantia nigra compacta of adult mice (Fig. 7). At three weeks post injection, we dissected the infected brain and quantified the loss of dopaminergic neurons by measuring TH immunoreactivity and Nissl staining in the substantia nigra pars compacta (Fig. 7). AAV-Rab35 T72A and AAV-Rab35 T72D caused 43.1 ± 11.7% and 27.7 ± 8.0% loss of TH-positive neurons and 47.1 ± 10.4% and 34.3 ± 4.8% loss of Nissl-positive neurons in the substantia nigra, respectively (Fig. 7b and c). By contrast, the number of TH- or Nissl-positive neurons in the substantia nigra was not affected by injection of AAV-hSyn-Rab35 WT. We verified that the expression levels of the different AAV-Rab35 constructs were similar in the ventral midbrain area containing the substantia nigra region (Additional file 2: Figure S2a) and we also confirmed the expression of AAV-hSyn-Rab35 WT, AAV-hSyn-Rab35 T72A, and AAV-hSyn-Rab35 T72D in TH-positive neurons of the substantia nigra after intracranial injection of AAVs (Additional file 2: Figure S2b). Together, these results suggest that dysregulation of Rab35 phosphorylation causes degeneration of dopaminergic neurons in vivo*.*

## Discussion

Previous studies on the subcellular localization and function of LRRK2 suggest that LRRK2 might play a role in intracellular vesicle trafficking. Several groups have reported physical, genetic or functional interactions between LRRK2 and a number of Rab GTPases, which serve as core regulators in perhaps all aspects of vesicle trafficking [18, 19, 26, 28, 29, 34]. Here, we performed a screening with 45 human Rab GTPases to examine phosphorylation of Rabs by LRRK2. Despite the fact that the switch II regions of Rab family members overlap significantly, we found that LRRK2 phosphorylated only a subset of Rab GTPases at a conserved threonine residue in the switch II region and that the extent of phosphorylation was quite variable. The strongly phosphorylated Rab proteins (Rab1, 3, 8, and 35) harbor highly conserved residues surrounding the LRRK2 site (Rab1a-T75, Rab3c-T94, Rab8a-T72, Rab35-T72) (see Fig. 2a). During revision of the current paper, Steger et al. [35] reported that LRRK2 directly phosphorylated Rab3, 5, 8, 10, 12, 29, 35, and 43 by performing a systematic proteomic analysis of HEK-293 cells overexpressing both LRRK2 and the Rab of interest. Our in vitro kinase assay revealed an overlapping, but not identical list of Rabs. In this study, we took a step forward and investigated the physiological consequence of such phosphorylation and found that dysregulation of the LRRK2 site in Rab1a, 3c, and 35 induced neurotoxicity in primary cortical neurons. Furthermore, intracranial injection of AAV expressing phosphomutants of Rab35 resulted in profound neurodegeneration in vivo*.* To the best of our knowledge, this study is the first to report that dysregulation of a direct substrate of LRRK2 causes neurodegeneration of dopaminergic neurons in the mammalian brain.

Rab GTPases interconvert between GTP- and GDP-bound states, which is regulated by their intrinsic molecular property and interaction with several types of specific effector proteins, including GEFs, GAPs, and GDIs. Phosphorylation of Rab might add an extra layer of regulation by altering the function of the Rab GTPase itself or that of its interacting partners [24, 29]. Several kinases can phosphorylate and regulate Rab family proteins: phosphorylation of Rab4 by p34^cdc2^ has been postulated to control its localization [36] and phosphorylation of Rab6c by protein kinase C to increase its affinity for GTP [37]. Rab5a, b, and c are differentially recognized by distinct kinases, for example, Rab5a by extracellular-regulated kinase 1 and Rab5b by p34^cdc2^ and LRRK2 [34, 38], and phosphorylation of Rab5b by LRRK2 on T6 increases GTP hydrolysis [34]. Rab8a has been suggested as a substrate of LRRK2, and the non-phosphorylatable Rab8a-T72A mutant exhibits increased binding to various regulatory proteins, including GDI1/2, a GEF protein Rabin-8, and a GAP protein TBC1D15 [29]. Our results also point to the same threonine site in Rab1a, 3c, 8a, and 35 being phosphorylated by LRRK2. When we monitored GDP/GTP-bound status in cells, we observed enhanced GTP-binding with phosphomimetic mutants and reduced GTP-binding with non-phosphorylatable mutants, for all the Rabs tested. Consistently, the level of Rabs bound to GTP was augmented by LRRK2-G2019S but was diminished by LRRK2-D1994A compared to wild-type LRRK2. At this point, it is not clear whether the phosphorylation regulated the intrinsic GTPase activity of Rab proteins or the binding with interacting partners, such as GAPs, GEFs or GDIs, which control GDP/GTP exchange. Nevertheless, our results suggest that LRRK2-induced phosphorylation results in inhibition of the GTP hydrolysis activity of Rab in cells. Alterations in nucleotide binding and hydrolysis induced by pathogenic LRRK2 might disrupt the balance between the cytosolic and membrane-bound pool of Rab proteins, and thereby cause defects in intracellular vesicle trafficking. GDP-bound Rab proteins are prone to extraction from membranes by Rab GDIs, and we found that the membrane-associated fraction of Rab35-T72A was reduced as compared to the wild-type. However, membrane-associated fraction of Rab35-T72D appeared to be similar to that of wild-type in our biochemical fractionation method, which enriched caveolin-1-positive plasma membrane fraction (Additional file 3: Figure S3). Further work is needed to determine if phosphorylation by LRRK2 stabilizes Rab proteins in their GTP-bound conformations and traps them in other membranous location(s).

Disruption of the LRRK2 site in Rab35 resulted in the most severe neurodegeneration compared to that induced by other Rabs, and phosphomutants of Rab35 were sufficient to cause degeneration of dopaminergic neurons in vivo. Notably, despite that phosphomimetic and phosphodeficient mutants showed opposite effects on GTP/GDP binding, such mutations induced neurodegeneration to a comparable degree, suggesting that neurodegeneration might be caused by impaired homeostasis, which would after the balance of Rab in its localization as well as activation and recruitment of downstream effectors. Rab35 has a single ortholog in human [31], and its activity is tightly controlled by at least four different DENN (differentially expressed in normal and neoplastic cells) family of GEFs and five different TBC (Tre2/Bub2/Cdc16) family of GAPs [39]. Rab35 has been shown to regulate diverse cellular processes, including endocytic recycling [40], exosome release [41], cytokinesis [42], and actin remodeling [30]. In the brain, Rab35 is expressed in multiple regions (Additional file 4: Figure S4), and in neurons, Rab35 seems to control neurite outgrowth during development [43–46] and synaptic vesicle trafficking [47]. However, little is known about its role in neurodegeneration. Interestingly, Rab35 was identified as a potential serum biomarker for PD through the analysis of proteomic profiles of PD patients, and Rab35 was also elevated in the substantia nigra obtained from multiple PD animal models, including MPTP-, rotenone-treated mice, and LRRK2-R1441C or -G2019S transgenic mice [48]. These results together with ours imply that impaired function of Rab35, perhaps in part through dysregulated phosphorylation, might contribute to neurodegeneration in PD. Further studies are needed to examine the extent, if any, to which the cellular processes known to be regulated by Rab35 are altered in LRRK2-associated neurodegeneration and PD pathogenesis.

Neurons are highly polarized cells and have extremely arborized cellular architecture, which imposes a substantial burden on the trafficking system. Thus, it is not surprising that intracellular trafficking deficits are associated with a number of neurodegenerative diseases, and PD is no exception. Dysfunction of Rab GTPases and impaired membrane traffic might contribute to the onset and progression of PD. Loss of *Rab39b* causes early-onset PD and *Rab29* resides in the *PARK16* non-familial risk locus [49–52]. Moreover, certain Rabs have been suggested to interact with and modulate the function of key pathogenic proteins, such as LRRK2, α-synuclein and PTEN-induced kinase 1 [17–19, 26, 29, 53–57]. Interestingly, overexpression of Rab1, 3a, and 8a, which we (this study) and others [29] suggest as LRRK2 substrates, could rescue α-synuclein-induced cytotoxicity in cell and animal model of PD [54, 55], implying that Rab GTPases might be involved in multiple pathways that play crucial roles in the pathogenesis of PD. Therefore, further study of the Rab GTPases will not only provide insight into how intracellular membrane dynamics are orchestrated, but also facilitate the identification of molecules or pathways that can serve as therapeutic targets for the treatment of PD.

## Conclusions

By performing in vitro LRRK2 kinase assays with forty-five human Rab GTPases, here we identify a subset of Rab GTPases, including previously identified as well as novel Rabs, such as Rab35, as authentic substrates of LRRK2. We provide evidence that phosphorylation of Rab GTPases by LRRK2 controls GDP/GTP exchange in cells and that dysregulation of Rab phosphorylation in the LRRK2 site causes neurodegeneration in primary neurons. Furthermore, we show that intracranial injection of AAV expressing phosphomutants of Rab35 into the substantia nigra induces profound degeneration of dopaminergic neurons in vivo. These results suggest that Rab GTPases might mediate LRRK2 toxicity in the etiology of PD.

## Additional files

Additional file 1: **Figure S1.** Immunostaining of cortical neurons transfected with Rab35 mutants. Representative images of embryonic day 15.5 cortical neurons expressing mutants (T72A, T72D or Q67Q) of GFP-conjugated Rab35. Neurons were fixed at one day after transfection and immunostained with anti-GFP, anti-smi312 (axonal marker), and anti-GFAP (astrocyte marker) antibodies. Scale bar, 100 μm. (PPTX 9498 kb)

Additional file 2: **Figure S2.** Expression of AAV-Rab35 WT and phosphomutants after intracranial injection of AAV. At one week after intracranial injection of AAV-eGFP (control), AAV-Rab35 WT, AAV-Rab35 T75A or AAV-Rab35 T75D into the substantia nigra, mouse brains were prepared for immunoblot (a) and immunohistochemistry (b). (a) Ventral midbrain was dissected from the AAV-injected hemisphere and the tissue was homogenized. Immunoblots were performed with anti-Rab35, anti-TH, and anti-actin antibodies. Exogenous and endogenous Rab35 are indicated by red and blue arrowhead, respectively. (b) Cryosections of AAV-injected brains including the substantia nigra region were immunostained with anti-GFP (green) and anti-TH (red) antibodies. Scale bar, 200 μm. (PPTX 3143 kb)

Additional file 3: **Figure S3.** Localization of Rab35 WT and phosphomutants. (a) HEK-293 cells were transfected with V5-tagged Rab35 WT or phosphomutants (T72A or T72D). At 48 h after transfection, cells were harvested and membrane fractionation was performed as described in Methods. Prepared membrane fractions were subjected to SDS-PAGE and immunoblotting with anti-caveolin-1 (plasma membrane marker), anti-EEA1 (early endosomal marker), anti-GOPC (golgi marker), and anti-Hsp90 (cytosol marker) antibodies. 2.5% of total lysates and 30% of membrane fractions were loaded for each immunoblot. (b) Quantification of Rab35 protein levels normalized against input level of Rab35. Data are mean ± SD (*n* = 3). * *p* < 0.05; one-way ANOVA followed by Dunnett’s multiple comparison post hoc test. (PPTX 1972 kb)

Additional file 4: **Figure S4.** Expression of Rab35 in adult mouse brain. Analysis of Rab35 protein expression by immunoblot in various brain regions of adult mouse. (PPTX 1820 kb)

## Abbreviations

AAV: Adeno-associated viral vectors
GAP: GTPase-activating protein
GDI: Guanine nucleotide dissociation inhibitor
GEP: Guanine nucleotide exchange factor
LRRK2: Leucine-rich repeat kinase 2
PD: Parkinson’s disease
