# Supplementary figures and images for: Dysregulated phosphorylation of Rab GTPases by LRRK2 induces neurodegeneration
Source: Mol Neurodegener. 2018 Feb 13;13:8. doi: 10.1186/s13024-018-0240-1 (PMC5811984; doi:10.1186/s13024-018-0240-1)

## Slide 1
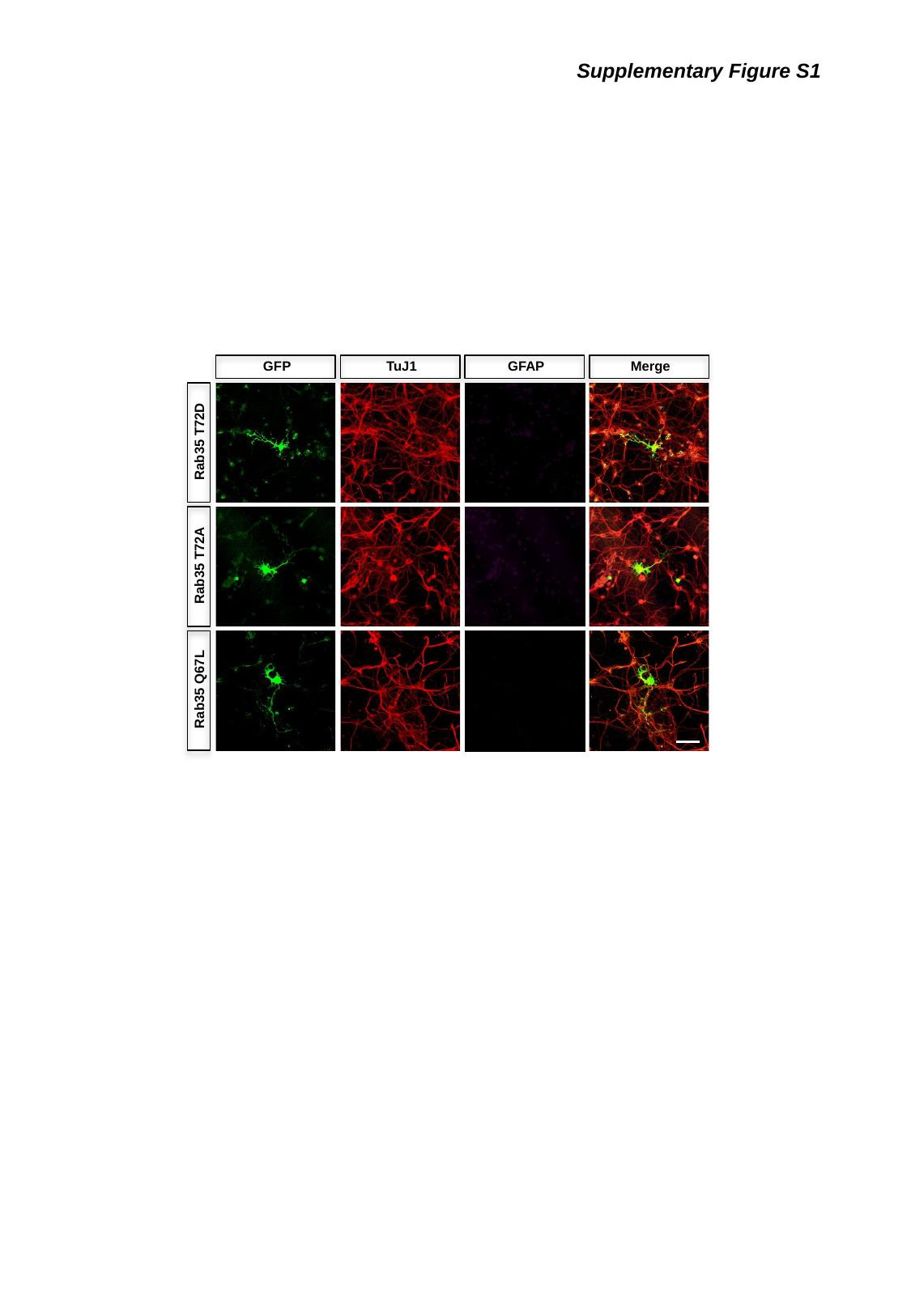

Supplementary Figure S1
GFP
TuJ1
GFAP
Merge
Rab35 T72D
Rab35 T72A
Rab35 Q67L

Supplement: Supplementary file 1 — Figure S1. Immunostaining of cortical neurons transfected with Rab35 mutants. Representative images of embryonic day 15.5 cortical neurons expressing mutants (T72A, T72D or Q67Q) of GFP-conjugated Rab35. Neurons were fixed at one day after transfection and immunostained with anti-GFP, anti-smi312 (axonal marker), and anti-GFAP (astrocyte marker) antibodies. Scale bar, 100 μm. (PPTX 9498 kb) [file 13024_2018_240_MOESM1_ESM.pptx]
