# Supplementary material for: Dysregulated phosphorylation of Rab GTPases by LRRK2 induces neurodegeneration
Source: Mol Neurodegener. 2018 Feb 13;13:8. doi: 10.1186/s13024-018-0240-1 (PMC5811984; doi:10.1186/s13024-018-0240-1)
Supplement: Supplementary file 2 — Figure S2. Expression of AAV-Rab35 WT and phosphomutants after intracranial injection of AAV. At one week after intracranial injection of AAV-eGFP (control), AAV-Rab35 WT, AAV-Rab35 T75A or AAV-Rab35 T75D into the substantia nigra, mouse brains were prepared for immunoblot (a) and immunohistochemistry (b). (a) Ventral midbrain was dissected from the AAV-injected hemisphere and the tissue was homogenized. Immunoblots were performed with anti-Rab35, anti-TH, and anti-actin antibodies. Exogenous and endogenous Rab35 are indicated by red and blue arrowhead, respectively. (b) Cryosections of AAV-injected brains including the substantia nigra region were immunostained with anti-GFP (green) and anti-TH (red) antibodies. Scale bar, 200 μm. (PPTX 3143 kb) [file 13024_2018_240_MOESM2_ESM.pptx]

## Slide 1
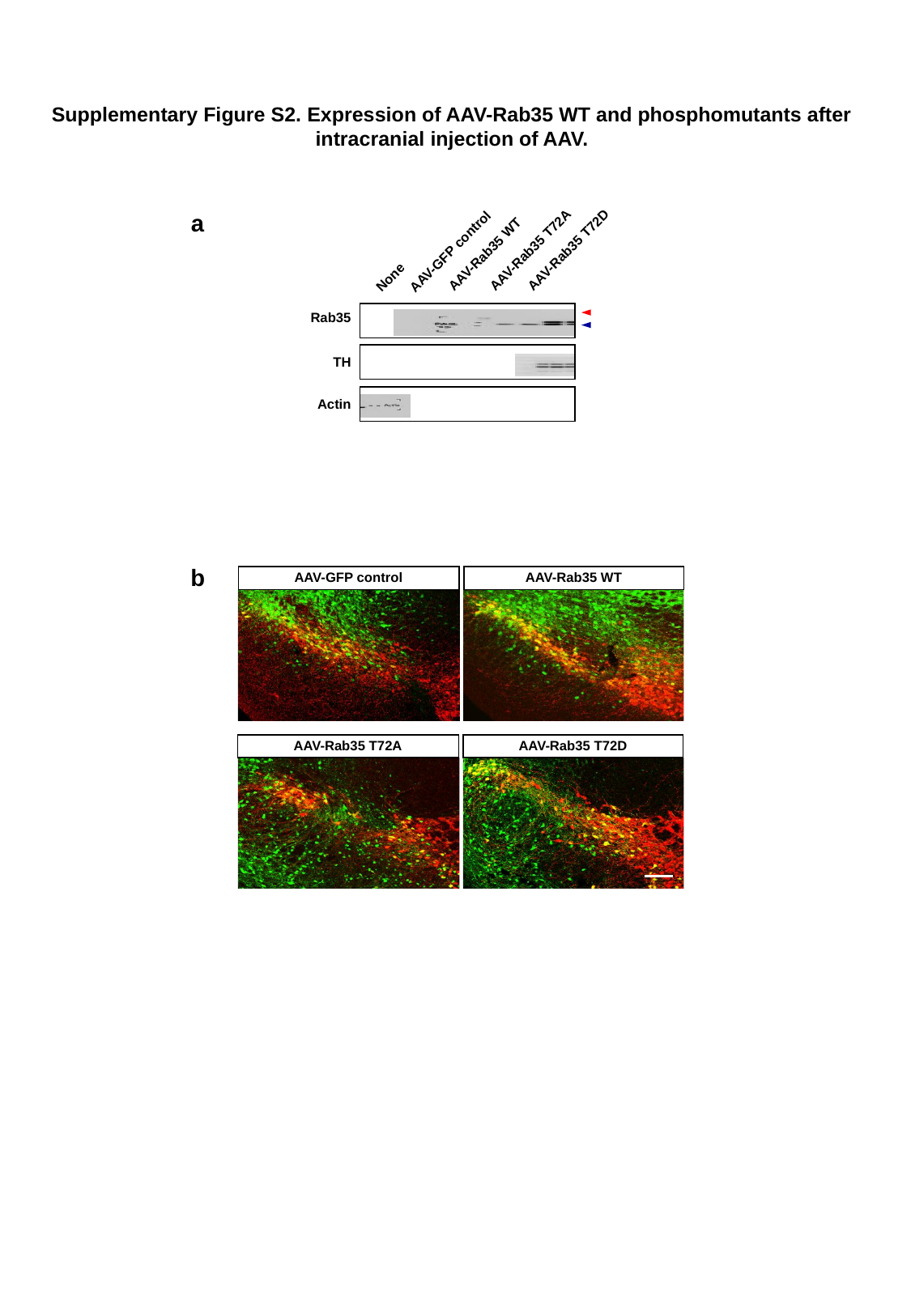

Supplementary Figure S2. Expression of AAV-Rab35 WT and phosphomutants after intracranial injection of AAV.
a
AAV-Rab35 T72A
AAV-Rab35 T72D
AAV-GFP control
AAV-Rab35 WT
None
Rab35
TH
Actin
b
AAV-GFP control
AAV-Rab35 WT
AAV-Rab35 T72A
AAV-Rab35 T72D
