# Supplementary material for: Dysregulated phosphorylation of Rab GTPases by LRRK2 induces neurodegeneration
Source: Mol Neurodegener. 2018 Feb 13;13:8. doi: 10.1186/s13024-018-0240-1 (PMC5811984; doi:10.1186/s13024-018-0240-1)
Supplement: Supplementary file 3 — Figure S3. Localization of Rab35 WT and phosphomutants. (a) HEK-293 cells were transfected with V5-tagged Rab35 WT or phosphomutants (T72A or T72D). At 48 h after transfection, cells were harvested and membrane fractionation was performed as described in Methods. Prepared membrane fractions were subjected to SDS-PAGE and immunoblotting with anti-caveolin-1 (plasma membrane marker), anti-EEA1 (early endosomal marker), anti-GOPC (golgi marker), and anti-Hsp90 (cytosol marker) antibodies. 2.5% of total lysates and 30% of membrane fractions were loaded for each immunoblot. (b) Quantification of Rab35 protein levels normalized against input level of Rab35. Data are mean ± SD (n = 3). * p < 0.05; one-way ANOVA followed by Dunnett’s multiple comparison post hoc test. (PPTX 1972 kb) [file 13024_2018_240_MOESM3_ESM.pptx]

## Slide 1
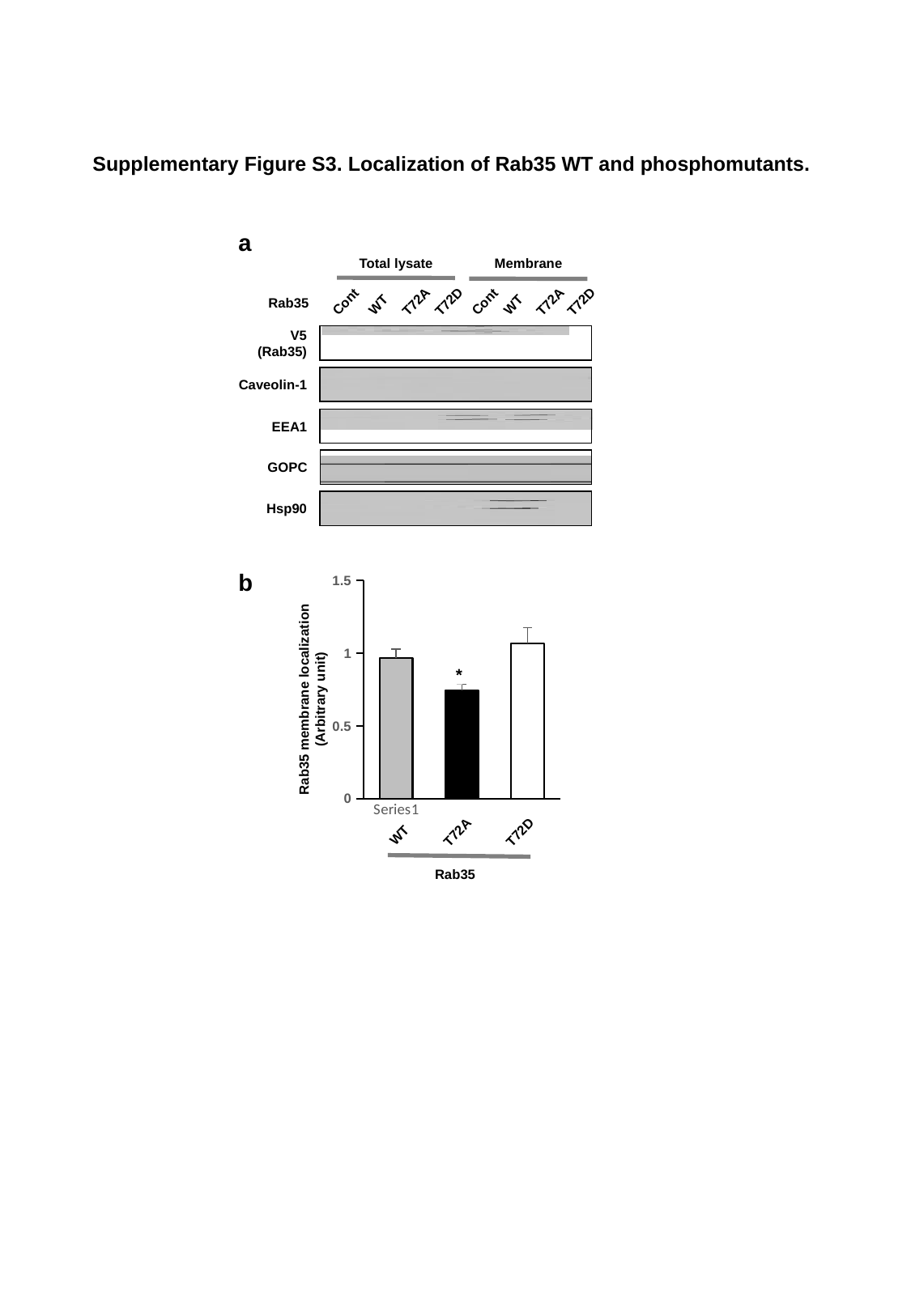

Supplementary Figure S3. Localization of Rab35 WT and phosphomutants.
a
Total lysate
Membrane
T72A
T72D
T72A
T72D
Cont
Cont
WT
WT
Rab35
V5
(Rab35)
Caveolin-1
EEA1
GOPC
Hsp90
b
### Chart
| Category | |
|---|---|
| | 0.9681915825950602 |
| | 0.7420519486755109 |
| | 1.0687127759733903 |*
Rab35 membrane localization
(Arbitrary unit)
T72A
T72D
WT
Rab35
