# Supplementary material for: Dysregulated phosphorylation of Rab GTPases by LRRK2 induces neurodegeneration
Source: Mol Neurodegener. 2018 Feb 13;13:8. doi: 10.1186/s13024-018-0240-1 (PMC5811984; doi:10.1186/s13024-018-0240-1)
Supplement: Supplementary file 4 — Figure S4. Expression of Rab35 in adult mouse brain. Analysis of Rab35 protein expression by immunoblot in various brain regions of adult mouse. (PPTX 1820 kb) [file 13024_2018_240_MOESM4_ESM.pptx]

## Slide 1
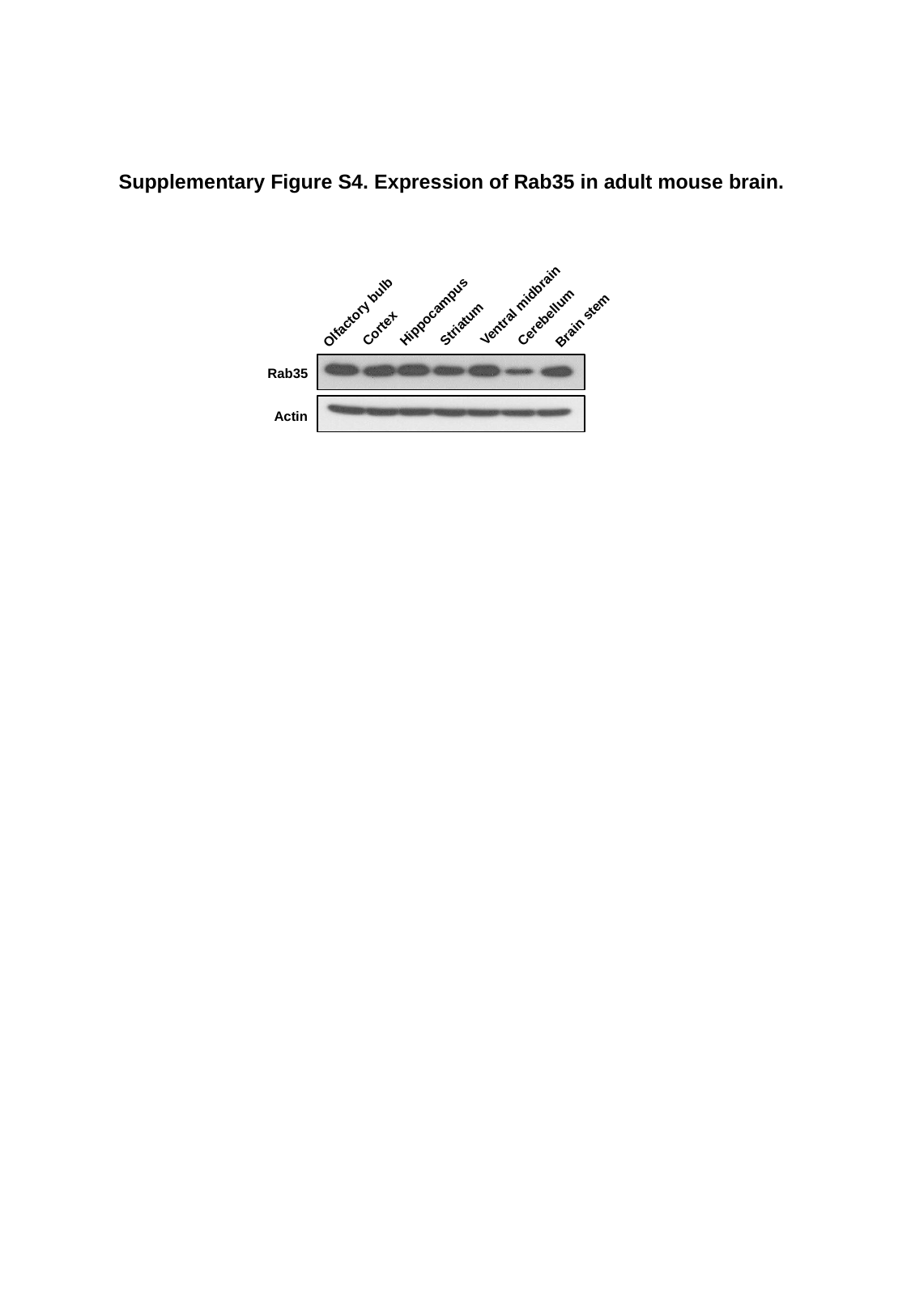

Supplementary Figure S4. Expression of Rab35 in adult mouse brain.
Ventral midbrain
Hippocampus
Olfactory bulb
Cerebellum
Brain stem
Striatum
Cortex
Rab35
Actin
